# Quantitative Imaging of Genetically Encoded Fluorescence Lifetime Biosensors

**DOI:** 10.3390/bios13100939

**Published:** 2023-10-19

**Authors:** Cong Quang Vu, Satoshi Arai

**Affiliations:** WPI Nano Life Science Institute, Kanazawa University, Kakuma-machi, Kanazawa 920-1192, Japan

**Keywords:** genetically encoded fluorescence lifetime biosensors, FLIM, FRET–FLIM, fluorescent proteins, quantitative imaging

## Abstract

Genetically encoded fluorescence lifetime biosensors have emerged as powerful tools for quantitative imaging, enabling precise measurement of cellular metabolites, molecular interactions, and dynamic cellular processes. This review provides an overview of the principles, applications, and advancements in quantitative imaging with genetically encoded fluorescence lifetime biosensors using fluorescence lifetime imaging microscopy (go-FLIM). We highlighted the distinct advantages of fluorescence lifetime-based measurements, including independence from expression levels, excitation power, and focus drift, resulting in robust and reliable measurements compared to intensity-based approaches. Specifically, we focus on two types of go-FLIM, namely Förster resonance energy transfer (FRET)–FLIM and single-fluorescent protein (FP)-based FLIM biosensors, and discuss their unique characteristics and benefits. This review serves as a valuable resource for researchers interested in leveraging fluorescence lifetime imaging to study molecular interactions and cellular metabolism with high precision and accuracy.

## 1. Introduction

The real-time measurement of specific molecules is a key approach for investigating and elucidating their biological roles and functions within cells. Fluorescence bioimaging has long been the cornerstone of biological research, enabling the visualization of cellular metabolites, molecular interactions, and dynamic processes. This has provided invaluable insights into various aspects of cellular function. Since the first demonstration of green fluorescent protein (GFP) in *Escherichia coli* and *Caenorhabditis elegans* in 1994 [1] and the initial report of a calcium (Ca^2+^) biosensor in 1997 [2], over 700 genetically encoded fluorescence biosensors have been developed for the detection of ions [3], metabolites [4], neurotransmitters [5], and physical factors, such as voltage [6], temperature [7], and molecular crowding [8]. Several of these biosensors sense their targets based on changes in their fluorescence intensity, leading to their classification as fluorescence intensity-based or intensiometric biosensors. However, traditional fluorescence intensity-based biosensors have several limitations, including dependence on the expression level and excitation power, sensitivity to pH changes, susceptibility to photobleaching, and the influence of focus drift during time-lapse imaging. These factors can lead to inconsistent and variable measurements, limiting their use in qualitative rather than quantitative assessments.

Ratiometric biosensors have been developed to address the intrinsic limitations of intensiometric biosensors. These sensors use two differently colored fluorescent proteins (FPs) and capitalize on Förster resonance energy transfer (FRET). FRET occurs when two FPs are in close proximity, allowing the transfer of energy from the donor FP to the acceptor FP through a nonradiative process. Instead of measuring the fluorescence intensity of a single FP, ratiometric biosensors can quantify the ratio of the fluorescence intensities of two FPs. This strategy effectively mitigates the issues associated with fluorescence intensity-based measurements, such as variations in biosensor concentration, excitation power fluctuations, photobleaching, and focus drift. Consequently, this yields a more reliable and quantifiable readout, thereby enhancing the precision of biological investigations. Although FRET-based biosensors have been shown to be invaluable in biological studies, they face a set of challenges. For instance, the spectral overlap between the donor and acceptor FPs can restrict the simultaneous use of multiple FRET-based biosensors in a single experiment because of interference from their emission spectra. An illustration of this can be found in a study in which three FRET-based biosensors for the kinase activities of protein kinase B (Akt), Src, and extracellular signal-regulated kinase (ERK) were simultaneously imaged [9]. This is in contrast to single-FP-based biosensors, in which five-color imaging has been demonstrated [10]. The broad excitation spectrum of the FRET-based biosensors can directly excite the FP acceptor, resulting in false positives. Additionally, differences in photobleaching and maturation rates between the donor and acceptor FPs can impact the FRET signal over time and affect the sensitivity and reliability of biosensors. These factors can lead to variability in the fluorescence ratio, requiring normalization against the initial ratio (Δ*R*/*R*), and thus limiting the ability for quantitative measurements.

Another type of ratiometric biosensor is an excitation ratiometric biosensor. These biosensors leverage the changes in equilibrium between the protonation and deprotonation states of tyrosine-based chromophore FPs such as wtGFP [11] and mKeima [12]. These changes are influenced not only by analyte binding but also by environmental changes, particularly changes in pH [13]. For imaging each ratio, two separate exposure times were required, using two different excitation wavelengths and recording fluorescence emission to quantify the ratio. Similar to FRET-based ratiometric biosensors, excitation ratiometric biosensors overcome the issues inherent to fluorescence intensity-based biosensors. However, the use of two distinct excitations with different exposure times renders the calibration of excitation ratios challenging for different samples and over time. This is because the excitation ratios can be affected by variations in the relative laser power and fluctuations at the two wavelengths, as well as by wavelength-dependent scattering.

On the other hand, genetically encoded fluorescence lifetime biosensors using fluorescence lifetime imaging microscopy (go-FLIM) offer a more robust and reliable alternative. By measuring the fluorescence lifetime rather than intensity, go-FLIMs are unaffected by changes in expression levels, excitation power, and focus drift and exhibit reduced pH sensitivity and minimized photobleaching. Importantly, the fluorescence lifetime values can be used directly for quantification without the need for normalization, rendering go-FLIM a powerful tool for highly accurate and reliable quantitative imaging. Moreover, because go-FLIMs are gene-encoded, they can be expressed as transgenes in target cells or tissues, significantly enhancing their applicability in biological research. There are two primary types of go-FLIMs: FRET–FLIM and single-FP-based FLIM biosensors. Both offer unique characteristics and benefits, and their use has the potential to advance our understanding of biomolecular interactions and cellular processes. In this review, we provide an overview of the principles, applications, and advancements of go-FLIMs. We highlight the advantages and discuss the challenges associated with their use. For the principles of FLIM, we recommend that the audience read other comprehensive reviews reported elsewhere [14,15]. Especially for detailed insights into FLIM analysis techniques, including curve fitting, phasor plots, and deconvolution methods, we recommend the comprehensive review by Datta et al. [15]. Our aim is to provide a resource for researchers interested in leveraging fluorescence lifetime imaging to study cellular processes with high precision and accuracy.

## 2. FRET–FLIM Biosensors

**Figure 1:**
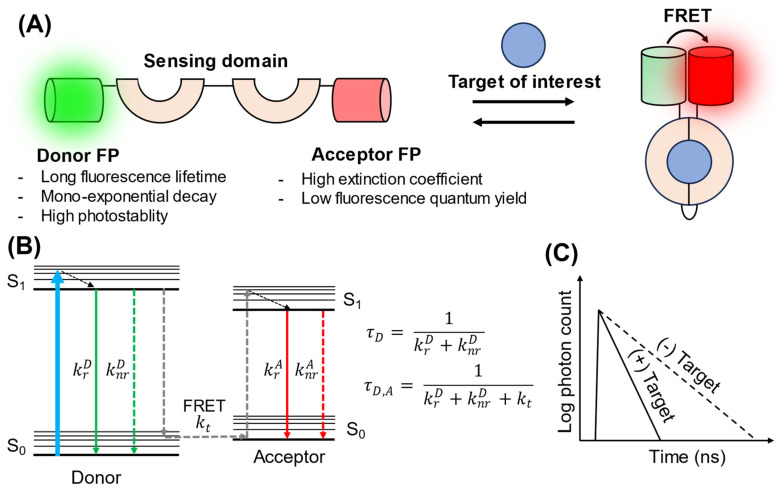
Schematic illustration of the design and sensing mechanism of FRET–FLIM biosensors. (**A**) Design of FRET–FLIM biosensors. A donor and acceptor FP are fused to a sensing domain that undergoes a conformational change upon binding to its target. This change brings the two FPs into close proximity, inducing FRET. (**B**) Jablonski diagram of FRET–FLIM [16]. S_0_ and S_1_ represent the ground state and excited states, respectively. Here, $k_{r}^{D}\;$is the radiative rate constant of the donor; $k_{nr}^{D}\;$is the nonradiative rate constant of the donor; $k_{t}$ is the energy transfer rate constant; $k_{r}^{A}$ is the radiative rate constant of the acceptor; $k_{nr}^{A}\;$is the nonradiative rate constant of the acceptor; $\tau_{D}$ is the fluorescence lifetime when only the donor is present; and $\tau_{D,A}$ is the fluorescence lifetime of the donor in the presence of an acceptor in the FRET pair. (**C**) Schematic representation of fluorescence decay in the presence and absence of the target. When FRET occurs, elevated *k_t_* results in a shortened fluorescence lifetime for the donor.

FRET–FLIM biosensors constitute a major class of go-FLIMs. They combine the advantages of FRET with the precision of FLIM to create a robust and versatile tool for biological investigations. FRET–FLIM exploits the energy transfer process between two proximal FPs, namely a donor and an acceptor FP. When these FPs are within a particular distance from one another (1–10 nm), typically brought together by molecular interactions or conformational changes induced by the target of interest, the energy of the excited state of the donor is nonradiatively transferred to the acceptor. This energy transfer process influences the fluorescence lifetime of the donor. Changes in the fluorescence lifetime can be quantified using FLIM, which provides an accurate measure of the presence or activity of the target (Figure 1).

Unlike traditional FRET-based biosensors, which often require normalization against the acceptor or donor fluorescence intensity, FRET–FLIM bypasses the requirement for such corrections by directly measuring the fluorescence lifetime of the donor. This feature significantly reduces experimental artifacts often associated with intensity-based measurements, such as autofluorescence, photobleaching, and excitation power variations. Additionally, the lifetime readout is independent of the donor fluorophore concentration. This allows for a more reliable and accurate measurement of molecular interactions, irrespective of the expression levels of the biosensor proteins. This feature is particularly advantageous for time-lapse imaging and single-molecule studies, in which the biosensor concentration can vary substantially. When combined with two-photon FLIM (2p-FLIM), FRET–FLIM biosensors have emerged as powerful instruments for in vivo studies that offer enhanced depth penetration.

FRET–FLIM biosensors have broad applications in biological research, including the quantification of intracellular metabolites, enzymatic activities, and protein–protein interactions (PPI) (Table 1). For instance, Epac-S^H189^ is a notable fourth-generation Epac-based FRET–FLIM biosensor tailored to detect the second messenger, cyclic adenosine monophosphate (cAMP). This advanced biosensor exhibited a notable improvement in its FLIM response, with a change of 1.48 ns [17]. By leveraging the capabilities of Epac-S^H189^, Harkes et al. pioneered screening methodologies that shed light on the distinct roles of 22 individual phosphodiesterases crucial for the breakdown of cAMP in HeLa cells [18]. Yasuda et al. further expanded upon this technology by introducing FRET–FLIM biosensors such as isozyme-specific translocation of C kinase (ITRACK) and isozyme-specific docking of C kinase substrate (IDOCKS) [19]. These sensors were designed specifically for the three classical protein kinase C (PKC) isozymes: PKC_α_, PKC_β_, and PKC_γ_. Moreover, they introduced Green–Camui_α_, which incorporated the mEGFP–REACh FRET pair, paving the way for precise measurements of calcium/calmodulin-dependent kinase II (CaMKII) activities within individual dendritic spines during the process of long-term potentiation [20]. FRET–FLIM biosensors offer significant advantages for studying PPI, such as the interaction between RhoC and RhoGDIγ. By fusing EYFP to RhoC and ECFP to RhoGDIγ, the fluorescence lifetime of ECFP decreased upon their interaction, as visualized through FLIM imaging [21].

Several considerations must be considered when designing FRET–FLIM biosensors. Ideally, the donor FP should possess a long fluorescence lifetime with mono-exponential fluorescence decay. The long fluorescence lifetime extends the dynamic range, whereas the simple decay kinetics simplify the determination of the distinct lifetime of the donor, both with and without FRET, using multiexponential fitting from the fluorescence decay histogram. In addition, the donor FP should exhibit high photostability and be non-photoconvertible. Examples of donor FPs suitable for FRET–FLIM include mTurquoise, with a fluorescence lifetime of 4.0 ns and a mono-decay curve [22], and NowGFP, with a fluorescence lifetime of 5.1 ns and single-exponential decay [23]. The acceptor FP, however, should have a high molecular extinction coefficient but an extremely low fluorescence quantum yield to avoid acceptor emission bleeding through the donor channel. To fulfill this purpose, several GFP-like nonfluorescent chromoproteins have been engineered to act as FRET acceptors, such as REACh [24], ShadowY [25], and Ultramarine [26]. The ideal “zero-emission” acceptor can also help to avoid spectral overlap, thereby facilitating multiplex imaging.

Despite these advantages, the use of FRET–FLIM biosensors is challenging. The design and optimization of FRET–FLIM biosensors require the careful selection of donor and acceptor FP pairs with suitable spectral properties and a well-defined spatial orientation to ensure efficient energy transfer. Notably, not all FRET-based biosensors can be used for FRET–FLIM. For instance, YCaMP3.60, a FRET-based Ca^2+^ biosensor, exhibits only a small lifetime change in response to Ca^2+^ [27]. Although direct excitation of the acceptor FP is not a concern in FRET–FLIM, variations in the maturation rate of the acceptor FP can influence energy transfer from the donor, which subsequently affects the fluorescence lifetime of the donor FP, causing variations between cells. This issue is more pronounced in certain cases of chromoprotein FRET acceptors, whose maturation times are relatively slow. For example, Ultramarine took 216 min to mature at 28 °C and 108 min at 37 °C [26], whereas EGFP required 14.9 min to mature [28]. In addition, the complexity and cost of FLIM instrumentation and analysis may deter its broader utilization. However, with ongoing advancements in FLIM technologies and FP engineering, FRET–FLIM biosensors hold significant promise for advancing our understanding of complex biological systems at the molecular level.

**Table 1:** A list of FRET–FLIM biosensors and their fluorescence lifetime responses.

Targets	Names	FRET Pairs	2p-FLIM	𝜏_free_ (ns)	𝜏_bind_ (ns)	Δ𝜏_(bind-free)_ (ns)	Ref.
Protein kinase A (PKA)	FLIM–AKAR	EGFP–cpsREACh	Yes	1.85	1.70	−0.15	[29]
	AKARet	sREAChet–EGFP	Yes	-	-	−0.21	[30]
	AKAR5	mEGFP–sREACh	Yes	-	-	0.20	[31]
	tAKARα	EGFP–cpsREACh	Yes	-	-	−0.26	[32]
						
Protein kinase B (PKB)/Akt	GFP–Akt–YFP	GFP–YFP		2.30	1.70	−0.60	[33]
							
Protein kinase C (PKC)	ITRACK_α_	mEGFP–mCherry	Yes	-	-	−0.22	[19]
	ITRACK_β_	mEGFP–mCherry	Yes	-	-	−0.18	[19]
	ITRACK_γ_	mEGFP–mCherry	Yes	-	-	−0.16	[19]
	IDOCKS_α_	mEGFP–mCherry	Yes	-	-	−0.31	[19]
	IDOCKS_β_	mEGFP–mCherry	Yes	-	-	−0.23	[19]
	IDOCKS_γ_	mEGFP–mCherry	Yes	-	-	−0.22	[19]
						
Aurora Kinase A	ShadowG–AURKA–mTQ2	ShadowG–mTQ2		-	-	0.15	[34]
	ShadowY–AURKA–mTQ2	ShadowY–mTQ2		-	-	0.15	[34]
							
Calcium/calmodulin-dependent kinase II (CaMKII)	Green–Camui_α_	mEGFP–REACh	Yes	1.67	2.08	0.41	[20]
	mRFP/GFP–Camui	mRFP–GFP		1.82	2.13	0.31	[35]
	Camui_α_–mRmC	mRuby2–mCherry_I202Y/T		-	-	0.10	[36]
						
Extracellular signal-regulated kinase (ERK)	EKARet	sREAChet–EGFP	Yes	-	-	−0.23	[30]
							
cAMP	^T^Epac^VV^	mTQ–^cp173^Venus-Venus		2.28	3.03	0.75	[22]
	^C^Epac^VV^	mECFP–^cp173^Venus-Venus		1.64	2.02	0.38	[22]
	Epac^SH189^	mTQ2–tdDark-^cp173^Venus		1.93	3.41	1.48	[17]
							
Ca^2+^	TN-L15	CFP–Citrine		2.36	1.9	−0.46	[37]
	mTFP–TnC-Cit	mTFP1–Citrine		2.51	2.18	−0.33	[37]
							
NAD+	ChemoD–NAD	ShadowG–HaloTag7		2.21	3.37	1.16	[38]
							
pH	pH–Lemon	mTQ2–EYFP	Yes	3.69 (pH 4.03)	2.48 (pH 7.01)	−1.21	[39]

## 3. Single-FP-Based FLIM Biosensors

**Figure 2:**
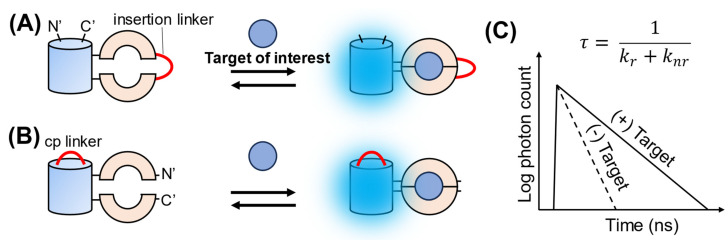
Schematic illustration of the design and FLIM response of single-FP-based FLIM biosensors. (**A**) Insertion type and (**B**) circular permutation (cp) type. For the insertion type, the sensing domain is split into two sections, each interconnected by an insertion linker. For the cp type, the FP is divided into two sections, each bridged by a cp linker. (**C**) Schematic illustration of the FLIM response. Upon binding to the target of interest, the non-radiative rate constant (*k_nr_*) may be altered (to be discussed later), causing changes in fluorescence lifetime (𝜏).

Another category of go-FLIMs is the single-FP-based FLIM biosensor. These are constructed by fusing an FP, either an insertion or circular permutation (cp), to a sensing domain (Figure 2). When the sensing domain binds its target, it undergoes a conformational change. This change affects the local environment surrounding the FP chromophore, leading to alterations in its fluorescence quantum yield. Because of the intrinsic relationship between the fluorescence quantum yield and lifetime, this alteration subsequently results in a change in the fluorescence lifetime. A notable advantage of these biosensors is their elimination of the need for donor–acceptor pairing, thus overcoming issues associated with spectral overlap, variable maturation rates, or potential interference between the paired proteins in FRET-based and FRET–FLIM biosensors. This simplified design often facilitates a more straightforward data interpretation, as changes in the fluorescence lifetime directly represent the interaction of the biosensor with its target molecule. Compared to fluorescence intensity-based biosensors, single-FP-based FLIM biosensors are not influenced by changes in expression level, power excitation, or focus drift, and they do not require corrections for photobleaching or normalization. Moreover, unlike fluorescence intensity-based biosensors, which can be significantly affected by pH owing to the protonation or deprotonation of the chromophore, single-FP-based FLIM biosensors exhibit reduced pH sensitivity [40]. Because single-FP-based FLIM biosensors require only a single excitation for imaging, they bypass challenges inherent in dual-excitation ratiometric biosensors, namely that two different wavelengths with distinct powers are required, and therefore complicating the consistency of ratio values across different samples and laboratories.

**Figure 3:**
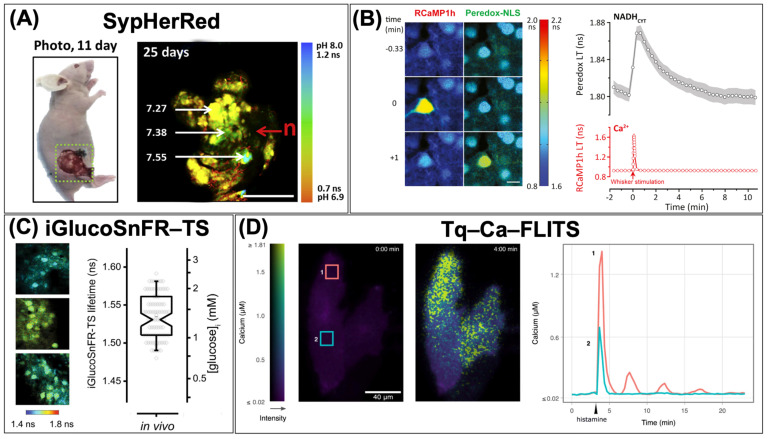
Cellular quantitative imaging using single-FP-based FLIM biosensors. (**A**) Quantification of pH in tumors by SypHerRed (reproduced with permission from Ref. [41]). (**B**) Measurement of cytosolic NADH/NAD+ ratio with Peredox and Ca^2+^ concentration with RCaMP1h in neuronal cells in response to whisker stimulation (reproduced with permission from Ref. [43]). (**C**) Mapping glucose concentration in cortical neurons of awake mice using iGlucoSnFR–TS. Fluorescence lifetime images (**left**) and the quantification of glucose concentration (**right**) (reproduced with permission from Ref. [44]). (**D**) Monitoring Ca^2+^ levels in endothelial cells with Tq–Ca–FLITS before and after stimulation with histamine. Fluorescence lifetime images (**left**) and quantifying Ca^2+^ concentration in ROI1 and ROI2 (**right**) (reproduced with permission from Ref. [27]).

Several single-FP-based FLIM biosensors have been developed for quantitative imaging of ions, metabolites, and neurotransmitters (Table 2). For instance, Shimolina et al. introduced SypHerRed, a red-color FLIM biosensor designed to quantify physiological pH, and applied it to quantify the absolute pH values in tumors (Figure 3A) [41]. The Yellen group developed a green-color FLIM biosensor named Peredox for quantifying the NADH/NAD+ ratio [42]. Together with RCaMP1h, a red-color Ca^2+^ indicator, they utilized Peredox to simultaneously measure the NADH/NAD+ ratio and Ca^2+^ concentration in the primary somatosensory cortex of an awake mouse in response to whisker stimulation (Figure 3B) [43]. Furthermore, the same group developed a FLIM-glucose biosensor, iGlucoSnFR-TS, for mapping glucose concentration in cortical neurons from awake mice (Figure 3C) [44]. Additionally, GRAB_ACh3.0_, a fluorescence intensity-based acetylcholine biosensor, has been effectively utilized for 2p-FLIM imaging [45]. Arai et al. recently developed qMaLioffG, a FLIM–ATP biosensor, and used it to distinguish different cell types and states based on ATP levels [40]. Moreover, innovations in cyan-color FLIM biosensors have recently emerged. For example, the Goedhart group developed a cyan-color FLIM–Ca^2+^ biosensor named Tq–Ca–FLITS, utilizing cp-mTurquoise2 (cp–mTQ2) [27]. With Tq–Ca–FLITS, they monitored Ca^2+^ level changes in endothelial cells before and after stimulation with histamine (Figure 3D). Koveal et al. also developed a cp–mTQ2-based FLIM–lactate biosensor [46]. One notable feature of these cp–mTQ2-based FLIM biosensors is their enhanced pH sensitivity, as reflected by their p*K*_a_ value of 4.7 [27]. This property ensures precise measurements, even in mildly acidic cellular environments, rendering them optimal tools for various biological applications where minor pH fluctuations play a pivotal role. Collectively, these innovative tools will facilitate multiplex FLIM imaging, allowing simultaneous quantification of various cellular metabolites.

Although single-FP-based FLIM biosensors have undeniable benefits, their development holds significant challenges. Notably, not every fluorescence intensity-based biosensor translates into a FLIM response. A key challenge arises from the unclear mechanism behind the fluorescence lifetime changes in single-FP-based FLIM biosensors. This is in contrast to FRET–FLIM biosensors, in which alterations in the donor fluorescence lifetime are attributed to energy transfer to the acceptor. Moreover, although the design framework for fluorescence intensity-based biosensors is well established, there is a notable absence of guiding papers or standardized methods for the design and screening of single-FP-based FLIM biosensors. However, considering the design similarities between single-FP-based FLIM and fluorescence intensity-based biosensors, strategies based on intensity-based biosensors can be applied to FLIM-based biosensors. For readers interested in diving deeper into design specifics, we recommend the comprehensive study by Nasu et al. [51]. Despite these challenges, single-FP-based FLIM biosensors stand out as pivotal tools that offer precise and accurate quantification of cellular activity.

## 4. Challenges and Limitations

Although go-FLIMs offer numerous advantages, their challenges and limitations must be considered for accurate data quantification and interpretation. FLIM is typically more expensive than fluorescence intensity-based measurements such as those conducted using epifluorescence microscopy. In addition, analyzing FLIM data requires specialized expertise, and the computational cost of FLIM analysis is often higher than that of intensity-based imaging. Data acquisition in FLIM is generally slower than in fluorescence intensity imaging because it requires the capture of a sufficient number of photons for accurate fitting to determine the fluorescence lifetime of each pixel. This often restricts the use of FLIM biosensors for the quantification of biological processes occurring on a timescale of seconds to minutes. However, recent advancements in FLIM microscopes have enabled FLIM images to be captured at video rates [52], rendering FLIM biosensors more applicable to biological studies.

Quantifying data from go-FLIM experiments requires proper calibration, as fluorescence lifetime can be influenced by various factors such as pH, temperature, local viscosity, and refractive index [53]. These factors can complicate the interpretation of changes in fluorescence lifetimes within biological systems. Therefore, researchers should consider these factors when quantifying and interpreting their data. Typically, calibration is carried out in a buffer solution using purified proteins. It is essential to prepare the calibration curves at various pHs and/or temperatures as closely as possible to the cellular experimental conditions. However, it is worth noting that buffer solutions do not precisely replicate the cellular environment due to the presence of numerous cellular macromolecules. To better mimic the crowded cellular environment, the buffer should include a molecular crowding reagent such as Ficoll PM70 at a concentration of 14% *w*/*w* [54]. Notably, calibration should also be performed using membrane-permeabilized cells, as this can best mimic the cellular condition. Several examples of in situ calibration can be found in the literature [27,40,44,46].

Additionally, autofluorescence from endogenous fluorophores can interfere with FLIM imaging. Common examples include NAD(P)H (𝜏_free_ = 0.4 and 𝜏_bound_ = 1–5 ns), FAD (𝜏_free_ = 2.3–2.9 and 𝜏_bound_ < 0.1 ns), and flavin mononucleotide (FMN; 𝜏 = 4.27–4.67 ns) [15]. Through this characteristic, label-free FLIM imaging for NAD(P)H and FAD has been developed to investigate metabolic heterogeneity in patient tumor-derived pancreatic organoids in response to cancer drug treatments [55]. Generally, these endogenous fluorophores are excited by blue or ultraviolet (UV) light. To mitigate this interference, excitation for go-FLIMs should be shifted more toward the red, away from the UV region. Consequently, red and near-infrared (NIR) go-FLIMs have emerged as potential candidates to overcome these challenges.

On the other hand, incident polarization can influence FLIM measurements, particularly in cases involving FRET such as FRET–FLIM. The orientation and polarization of the excitation light can affect the measured fluorescence lifetime, as they influence the energy transfer between donor and acceptor fluorophores in FRET pairs [56]. Additionally, the selection of the excitation wavelength is another consideration. It should align with the absorption spectra, whether in one- or two-photon FLIM, of the biosensor to achieve an optimal fluorescence signal. Moreover, the intensity of the excitation light can impact both the quality of the acquired data and the potential photobleaching or phototoxicity of biological samples. Although FLIM is generally independent of the excitation power and photobleaching, the selection of an appropriate excitation power level is essential to balance the signal-to-noise ratio and ensure sample safety.

In addition, the transfection of go-FLIM and genetically encoded biosensors presents a significant challenge, particularly when working with primary and differentiated cells. This challenge has impeded the broader applicability of genetically encoded biosensors in biological research. To address this issue, various innovative approaches have been developed. Notably, Kreitz et al. recently introduced a method for delivering purified proteins into eukaryotic cells by employing a bacterial contractile injection system [57]. They successfully demonstrated the efficient delivery of Cas9 protein into both human cells and mice, achieving 100% delivery efficiency. This revolutionary method holds the potential to facilitate the delivery of go-FLIM-purified proteins into difficult-to-transfect cells and animal models, significantly expanding the applicability of go-FLIM across various research fields.

## 5. Future Perspectives and Conclusion

### 5.1. Strengthening in the Design and Screening Methods

**Figure 4:**
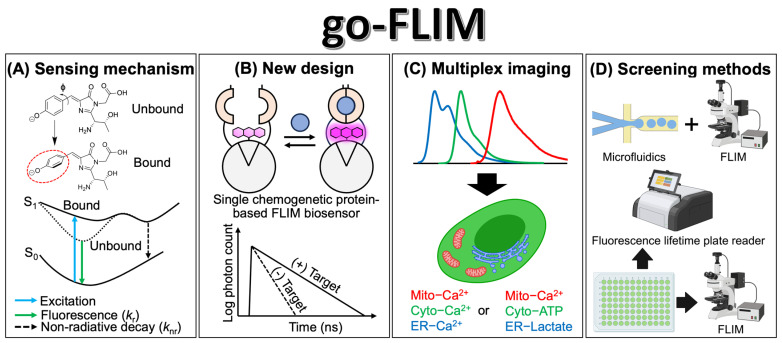
The promising future of go-FLIM development. (**A**) A schematic representation illustrating the proposed sensing mechanism of single-FP-based FLIM biosensors. A red circle indicates the rotation of chromophore. (**B**) Conceptual design of a single chemogenetic protein-based FLIM biosensor and its FLIM response. (**C**) Representation of multiplex imaging employing go-FLIMs that target various analytes across different organelles such as the mitochondria (mito), cytoplasm (cyto), and endoplasmic reticulum (ER). (**D**) Introduction of methodologies for the screening of go-FLIM. Illustrations were created with BioRender.com.

The advancement of go-FLIMs, particularly single-FP-based FLIM biosensors, is promising for significant innovations in the future. Central to this evolution is understanding the sensing mechanism behind the fluorescence lifetime changes in these biosensors (Figure 4A). Single-FP-based FLIM biosensors may not strictly adhere to the exchange mechanism between the protonated and deprotonated states of tyrosine-based chromophores. We speculate that binding to the target may increase the degree of freedom surrounding the chromophore. This could, in turn, lead to changes in the nonradiative pathway, resulting in alterations of the fluorescence lifetime as observed in mCherry [58]. However, for a deeper understanding of this mechanism, extensive collaboration is required across research fields. Techniques such as X-ray crystallography, nuclear magnetic resonance (NMR), and cryogenic electron microscopy (cryo-EM) can help resolve the atomic structure of a chromophore in both its bound and unbound states. In addition, molecular dynamics simulations can provide insights into this mechanism. Once the sensing mechanism is elucidated, it will open avenues for the rational design and conversion of fluorescence intensity-based biosensors into FLIM biosensors.

The use of advanced fluorophores with long fluorescence lifetimes and high photostabilities can significantly enhance the dynamic range and robustness of the measurements. Lumazine-binding protein (LUMP) shows promise in this regard. When non-covalently bound to ribityl-lumazine, LUMP emitted cyan-colored fluorescence with the longest average fluorescence lifetime of 13.6 ns among genetically encoded FPs [59]. Leveraging LUMP in FLIM biosensor applications can potentially amplify their dynamic range. Additionally, the adoption of synthetic fluorophores may improve molecular brightness and photostability. Compared with traditional FPs, synthetic fluorophores, such as rhodamine, often exhibit superior physical properties, such as high molecular extinction coefficients, fluorescence quantum yields, and high photostability [60]. Recently, Hellweg et al. introduced an innovative strategy that employed a rhodamine-labeled HaloTag as an acceptor for donor FP, thus creating a new generation of chemogenetic FRET-based biosensors [38]. Significant dynamic ranges in the fluorescence ratio were achieved, as seen with Ca^2+^ (36.1-fold), ATP (12.1-fold), and NAD+ (34.7-fold), especially after merging the chromoprotein ShadowG with a far-red rhodamine-labeled HaloTag on an NAD+ sensing domain to develop a far-red NAD+ FRET–FLIM biosensor with a 1.2 ns dynamic range [38]. This suggests the potential for engineering fluorescently labeled HaloTags for use in FRET–FLIM biosensors. Moreover, recent advances in biosensor development have highlighted the circularly permuted HaloTag (cp-HaloTag) [61] and SNAP-tag (cp-SNAP-tag) [62]. These developments suggest that fluorophores linked to the cp-HaloTag or cp-SNAP-tag, similar to cp-FPs, are responsive to local environmental changes. We hypothesize that leveraging this characteristic would advance the development of a novel class of chemogenetic protein-based FLIM biosensors (Figure 4B). For instance, Farrants et al. recently reported a chemigenetic calcium biosensor called WHaloCaMP1a, which leveraged the near-infrared-emitting dyes JF_669_ and HaloTag [63]. WHaloCaMP1a exhibited a fluorescence lifetime change of 2.1 ns upon binding to Ca^2+^. The significant advantage of employing synthetic fluorophores lies in their tunable emission wavelengths across the spectrum from blue to far-red [60], offering a versatile toolbox for multiplex imaging. However, the use of exogenous fluorophores may introduce some limitations, such as the commercial availability of certain fluorophores and challenges related to cell permeability and labeling efficiency. Nevertheless, the development of multicolored FLIM biosensors enables simultaneous quantification of the same analyte across various organelles or multiple analytes within the same cellular environment (Figure 4C).

The development of screening methods for go-FLIMs is crucial (Figure 4D). Koveal et al. recently introduced a high-throughput screening technique to expedite the development and optimization of soluble genetically encoded fluorescent biosensors [46]. This innovative method harnesses droplet microfluidics combined with automated 2p-FLIM. Briefly, individual DNA molecules from a mutant library were encapsulated in semipermeable gel-shell beads (GSBs). These GSBs are permeable to analytes with molecular weights less than 2 kDa. The biosensors were then expressed using an in vitro-coupled transcription/translation (IVTT) system. Subsequently, GSBs were organized on a glass coverslip for automated fluorescence lifetime imaging and analysis. The GSBs displaying the desired biosensor attributes were selected using a micropipette. The genotype of the biosensor was determined from the DNA beads using PCR, followed by identification through DNA sequencing. This method has a screening capability of approximately 10,000 variants per week. However, their complexity may limit their global accessibility. Alternatively, a more straightforward approach is to use a fluorescence lifetime plate reader for screening [64]. This approach has already been successfully used for the high-throughput screening of drug molecules specific to sarco/endoplasmic reticulum calcium ATPase (SERCA) [65] and ryanodine receptor type 2 (RyR2) channels [66]. On the other hand, direct screening using a FLIM microscope equipped with an automated system has shown its value in such applications. For example, Guzmán et al. developed an automated FLIM measurement setup by integrating a motorized stage with a FLIM microscope. This system allowed for FLIM measurement of the inner 60 wells of a 96-well plate in less than 20 min [67]. However, despite these advancements, there is still a need for the development of a high-throughput automated system for screening go-FLIMs. Achieving this goal requires advancements in both automation technology and high-speed FLIM measurements. Notably, Leica has recently introduced the SP8 FALCON microscope for high-speed FLIM measurements [52]. Incorporating a motorized microscope stage into the SP8 FALCON would drastically increase the screening speed. Moreover, direct screening in mammalian cells offers the most accurate representation of the actual cellular conditions when evaluating go-FLIMs. Lin et al. recently introduced a functional imaging-guided cell selection platform for screening far-red genetically encoded fluorescent calcium indicators [68]. In this approach, a mutant library of the biosensor, fused with a photoactivated PAmCherry, was transfected into HEK293T cells. Subsequently, the cells were exposed to the ionomycin drug to induce a Ca^2+^ response while capturing the fluorescence signal. The cells demonstrating a substantial change in fluorescence were labeled through the photoactivation of PAmCherry. These labeled cells were then isolated using fluorescence-activated cell sorting (FACS) to determine the DNA sequence of the biosensor. Remarkably, this method enabled the screening of 10^4^–10^5^ variants at a time. This approach can also be applied to the screening of go-FLIMs.

### 5.2. Potential Applications and Impact on Biological Research

The advent of go-FLIMs signals a paradigm shift in our approach toward understanding biological processes, emphasizing quantitative analysis over qualitative approximations. Go-FLIMs allow the precise measurement of cellular complexities, such as capturing the concentration of metabolites or detailing the subtle dynamics of protein interactions. Transitioning from a relative understanding to a more definitive and quantitative perspective will facilitate innovative applications and discoveries in various biological fields.

One example is cellular metabolism. Whereas conventional qualitative methods only provide relative changes in metabolites over time, go-FLIMs provide quantitative insights into metabolic fluxes, offering an in-depth view of cellular energy dynamics. For instance, Díaz-García and colleagues utilized the iGlucoSnFR–TS [44], a FLIM-glucose biosensor, to quantify the glucose concentration in dentate granule neurons in hippocampal slices and found that the intracellular glucose levels were ~20% less than the extracellular levels. Furthermore, they quantified intracellular glucose concentrations of 0.7–2.5 mM in individual neurons of awake mice. Such detailed observations, which are imperceptible using traditional fluorescence intensity-based biosensors, underscore the power of go-FLIMs.

FRET–FLIM technology stands out as a transformative pharmacological tool that offers quantitative insights into drug-target interactions. This technology empowers researchers to precisely assess binding affinities, calculate rate constants, and identify potential off-target effects of drug candidates, thereby facilitating drug development. An illustration of this capability is seen in the discovery of several potential small-molecule effectors of SERCA using a FRET–FLIM SERCA biosensor [65]. By fusing GFP–RFP as a FRET pair with specific sites on SERCA, the structural changes in SERCA induced by small-molecule effectors can be detected using a fluorescence lifetime plate reader. With the high precision and robustness of the FRET–FLIM measurements, they identified six inhibitory compounds out of the 727 tested. Moreover, this approach has been expanded to drug screening for other targets, such as tumor necrosis factor receptor 1 (TNFR1) [69], ryanodine receptor (RyR) [70], and RyR2 [66] calcium channels. Through its achievements, FRET–FLIM promises a brighter and more efficient future in drug development and is poised to uncover a wealth of potential therapeutics for diverse target molecules.

### 5.3. Conclusions

The impact of go-FLIMs in biological research cannot be overstated. By offering a lens of quantification, they have the potential to unveil the layers of previously obscured cellular processes. Potential applications span basic research, elucidating the mysteries of cellular machinery to translational processes and heralding a new era of precision medicine and targeted therapies. In addition, as FLIM technology continues to evolve rapidly, go-FLIM is anticipated to adapt, refine, and diversify. This will provide greater resolution and broader applications, effectively catalyzing multidisciplinary collaborations among biologists, chemists, physicists, and clinicians. The universal utility of this technology across various research areas underscores its indispensability. 

## Figures and Tables

**Table 2:** Reported single-FP-based FLIM biosensors for the detection of various targets.

Targets	Names	FP Reporters	2p-FLIM	𝜏_free_ (ns)	𝜏_bind_ (ns)	Δ𝜏_(bind-free)_ (ns)	Ref.
NAD+/NADH	Peredox	cpT–Sapphire	Yes	2.63	1.87	−0.76	[42]
							
Glucose	iGlucoSnFR–TS	cpT–Sapphire	Yes	1.40	1.78	0.38	[44]
							
Lactate	LiLac	cp–mTQ2	Yes	3.00	1.80	−1.20	[46]
							
ATP	qMaLioffG	Citrine		2.57	1.49	−1.08	[40]
							
Acetylcholine	GRAB_ACh3.0_	cpGFP	Yes	3.34	3.51	0.17	[45]
							
Histidine	FHisJ	cpYFP		2.80	1.60	−1.20	[47]
							
Ca^2+^	Tq–Ca–FLITS	cp–mTQ2		1.40	2.78	1.38	[27]
	CatchER	EGFP		2.18	2.61	0.43	[48]
	RCaMP1h	cp–mRuby	Yes	-	-	1.10	[43]
							
pH	pHRed	mKeima–A213S	Yes	1.72 (pH 5)	2.12 (pH 8)	0.40	[13]
	SypHerRed	cp–mApple	Yes	0.72 (pH 6.9)	1.05 (pH 7.7)	0.33	[41]
	SypHer3s	cpYFP	Yes	1.20 (pH 6.5)	2.30 (pH 9.5)	1.10	[49]
							
H_2_O_2_	Hyper3	cpGFP		1.29	0.92	−0.37	[50]
